# The role of computer-assisted systems for upper-endoscopy quality monitoring and assessment of gastric lesions

**DOI:** 10.1093/gastro/goab008

**Published:** 2021-03-12

**Authors:** Daniela Cornelia Lazăr, Mihaela Flavia Avram, Alexandra Corina Faur, Ioan Romoşan, Adrian Goldiş

**Affiliations:** 1 Department V of Internal Medicine I, Discipline of Internal Medicine IV, “Victor Babeș” University of Medicine and Pharmacy Timișoara, Romania,Timișoara, Romania; 2 Department of Surgery X, 1st Surgery Discipline, “Victor Babeș” University of Medicine and Pharmacy Timișoara, Romania, Timișoara, Romania; 3 Department I, Discipline of Anatomy and Embriology, “Victor Babeș” University of Medicine and Pharmacy Timișoara, Romania, Timișoara, Romania; 4 Department VII of Internal Medicine II, Discipline of Gastroenterology and Hepatology, “Victor Babeș” University of Medicine and Pharmacy Timișoara, Romania, Timișoara, Romania

**Keywords:** artificial intelligence, computer-assisted diagnosis, gastric cancer, premalignant gastric lesion, upper-endoscopy quality control

## Abstract

This article analyses the literature regarding the value of computer-assisted systems in esogastroduodenoscopy-quality monitoring and the assessment of gastric lesions. Current data show promising results in upper-endoscopy quality control and a satisfactory detection accuracy of gastric premalignant and malignant lesions, similar or even exceeding that of experienced endoscopists. Moreover, artificial systems enable the decision for the best treatment strategies in gastric-cancer patient care, namely endoscopic vs surgical resection according to tumor depth. In so doing, unnecessary surgical interventions would be avoided whilst providing a better quality of life and prognosis for these patients. All these performance data have been revealed by numerous studies using different artificial intelligence (AI) algorithms in addition to white-light endoscopy or novel endoscopic techniques that are available in expert endoscopy centers. It is expected that ongoing clinical trials involving AI and the embedding of computer-assisted diagnosis systems into endoscopic devices will enable real-life implementation of AI endoscopic systems in the near future and at the same time will help to overcome the current limits of the computer-assisted systems leading to an improvement in performance. These benefits should lead to better diagnostic and treatment strategies for gastric-cancer patients. Furthermore, the incorporation of AI algorithms in endoscopic tools along with the development of large electronic databases containing endoscopic images might help in upper-endoscopy assistance and could be used for telemedicine purposes and second opinion for difficult cases.

## Introduction

Starting from the middle of the twentieth century, the idea of artificial intelligence (AI) has attracted the attention of scientists in an attempt to augment the human brain’s capacity to solve different problems, from the daily routine to the most complex tasks, with the aid of computers [1]. In time, this concept has spread into different domains of activity. Currently, AI has begun to work its way into our everyday lives and we are counting on a multitude of available devices to make our lives easier, e.g. to wake us in the morning, forecast our weather, and help us to drive our cars. We have the opportunity to use translation and linguistic programs, unlock our mobile phones using facial recognition, access different applications, benefit from automation of our homes to make our environment more comfortable, and more [2].

The term *artificial intelligence (AI)* refers to the ability of a computer program to mimic the human brain by simulating certain cognitive functions and intelligent behaviors, such as the capacity to “learn” and “solve problems” [3, 4]. The concept of *machine learning (ML)* refers to an AI field whereby a computer system can automatically build mathematical algorithms based on input training data, subsequently being able to predict and generate decisions in uncertain circumstances without specific programming [4]. ML includes both handcrafted and deep-learning algorithms. In the handcrafted models, features are manually engineered by the data scientist; an algorithm is trained to perform classification of features and to recognize the class of a new image [5].

**Figure 1.**
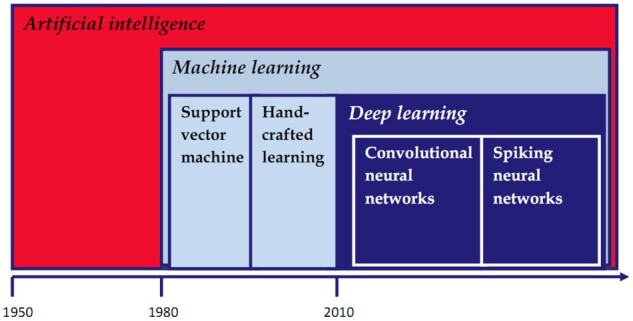
Artificial intelligence: leading concepts.

*Deep learning (DL)* refers to a subcategory of ML techniques constructed from multiple-layered *artificial neural-network* algorithms that shows similarities with the human brain, with the ability of automatically extracting and learning specific features of the training data set to elaborate a concrete result. The *convolutional neural network (CNN)* represents a class of deep neural networks that is most commonly applied to analysing visual imagery including medical images. They are composed of convolutional and pooling layers, with the role of extracting distinct features and fully connected layers with the ability to perform the overall classification. *Spiking neural networks* represent a type of artificial network that resembles more closely natural neural networks; their operating model includes the concept of time [4] (Figure 1).

Recent studies have been developed using AI to improve the quality of endoscopic procedures with the assistance of AI [3]; the detection of early neoplasia in Barrett’s esophagus [6, 7]; the diagnosis of early gastric cancer (EGC) and precursor lesions [8]; the detection of *Helicobacter pylori* infection [9] and gastric polyps [10]; the identification of small-bowel angioectasias, bleeding, polyp/ulcer/cancer, or hookworms on capsule endoscopy [11–13]; the dysplasia in intraductal papillary mucinous tumors [14]; and many other conditions. A large number of studies have also focused on improving the adenoma-detection rate at colonoscopy and on the distinction between adenomatous and hyperplastic polyps [15–20].

The aim of the paper is to familiarize clinicians with the continuously evolving field of AI and the way in which this modern technology will have an impact on endoscopy performance and its development in the near future in the daily clinical setting. The article presents an overview of the literature regarding the value of AI in monitoring the quality of esogastroduodenoscopy (EGD), whilst providing a comprehensive analysis of the existing literature that assesses the potential benefits and limits in the detection of premalignant and malignant gastric lesions and the prediction of gastric-cancer-invasion depth by adding different types of DL algorithms to both conventional and advanced endoscopic techniques.

## Methods

All English-language literature published in the last 15 years, before July 2020, was searched by assessing the PubMed electronic database. The keywords used for our research purposes were “gastric cancer,” “gastric neoplasm,” “gastric precancerous lesion,” “Helicobacter pylori infection,” “gastric polyp,” “esogastroduodenoscopy,” “upper endoscopy quality control,” “artificial intelligence,” “machine learning,” “deep learning,” “convolutional neural network,” “detection,” “diagnosis.” Furthermore, we searched to identify clinical studies involving AI for the endoscopic evaluation of gastric premalignant conditions and gastric cancer using the ClinicalTrials.gov database, University Hospital International Network–Clinical trial Registry (UMIN-CTR) and Chinese Clinical Trial Registry.

## Brief description of the main applications of AI in upper endoscopy

*Frame-detection task—*AI detects individual frames in a sequence of images containing suspicious lesions that that require more detailed examination, such as the detection of images containing gastric polyps during endoscopy; the role of this task is to prevent the endoscopist from missing a lesion [10].

*Object-detection task—*During endoscopy, AI is able to recognize a region of interest (ROI), such as a dysplastic area [5].

*Classification task—*AI categorizes the identified lesions into different classes such as neoplastic vs non-neoplastic, e.g. gastric cancer vs gastritis. Additionally, this task might involve assessment of the invasion depth of a malignant gastrointestinal (GI) lesion, such as in stomach cancer [21, 22].

*Segmentation task—*AI outlines the border of a detected lesion, making an accurate differentiation between pathological and healthy tissue. This delineation task was satisfactorily implemented for delineation of early gastric cancers on still images [23, 24].

**Figure 2.**
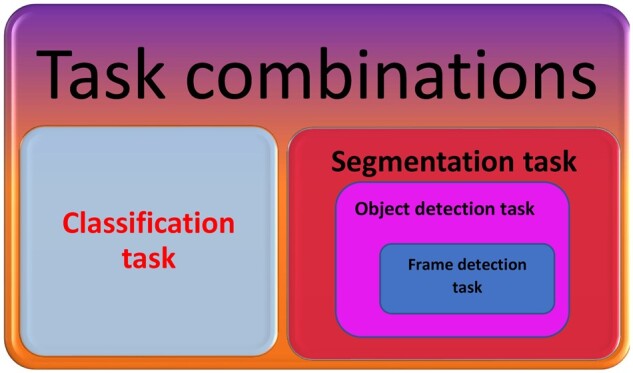
The main applications of artificial intelligence (AI) in upper endoscopy.

*Task combinations—*AI can combine the previously mentioned tasks into one work-flow, e.g. the detection and classification of a specific lesion followed by delineation of its border [10, 25] (Figure 2).

Application of AI in endoscopy consists of training a computer algorithm to fulfill a specific task, such as detection or characterization of a particular lesion. The training procedure takes place through exposure on big data sets, such as a large number of predefined video frames containing the examined lesion. These algorithms extract and assess specific features from a multitude of video frames, such as texture or color-hue differences, microsurface/microvascular/pit patterns, and many other features detectable using either white-light endoscopy (WLE) or other endoscopic technological advances such as high magnification, chromoendoscopy (CE), narrow-band imaging (NBI), linked color imaging (LCI), or endocytoscopy [26].

**Figure 3.**
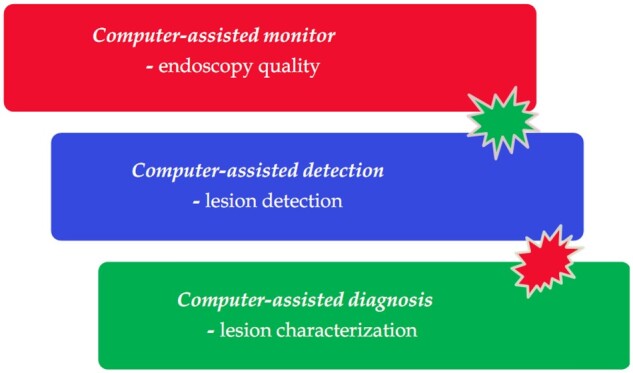
Categories of AI systems in the gastrointestinal-endoscopy field.

The great variety of ML technologies offer the ability to cover a large spectrum of functions and fulfill many tasks in the GI endoscopy field. Two main categories of AI systems exist, namely *computer-assisted detection (CADe)* [3, 18, 27, 28] with the task of lesion detection and *computer-assisted diagnosis (CAD)* [3, 22, 24, 29] with the task of lesion characterization, which also enables performance of “optical biopsy.” Furthermore, several systems are designed to offer technical assistance for improved endoscopy performance and quality, and are defined as *computer-assisted monitor (CADm)* systems [30–32] (Figure 3). Still other systems are constructed to offer assistance during endoscopic therapeutic procedures, e.g. assuring better delineation of an early esophageal tumor for complete endoscopic resection [33, 34].

## Principal applications of AI for upper-endoscopy quality-control monitoring and assessment of gastric premalignant and malignant lesions

### EGD quality-control improvement: computer-assisted monitor (CADm) systems

High-quality gastroscopy is mandatory for obtaining improved diagnostic accuracy and prognosis for patients [35]. Because of the marked differences in EGD performance among endoscopists (which lead to variation in the gastric premalignant- and malignant-condition detection rates and missed lesions [36]), numerous guidelines of different professional societies have developed evidence-based performance measures for upper endoscopy [37–39] and proposed standardized protocols to map the entire stomach [40, 41]. However, these protocols are not always followed, and therefore it is essential to develop practical modalities that can aid implementation in the daily routine [42].

WISENSE is a real-time quality-improvement system in endoscopy that was developed using the methods of deep CNN and deep reinforcement learning (DRL)—another component of DL that became known because of the Go game (2016). This system combines high perception capacity in visual tasks and decision-making capacity to address complex dynamic situations [43]. A single-center randomized–controlled trial (trial registration number ChiCTR1800014809) [30] was elaborated to test the ability of WISENSE to reduce the rate of blind spots (unseen areas) during upper endoscopy. WISENSE was based on the systematic screening protocol for the stomach, which was proposed as a minimum required standard for gastroscopy. For training of the network, 12,220 *in vitro*, 25,222 *in vivo*, and 16,760 EGD images from >3,000 patients (Dataset 1) were used and, for learning and classifying gastric sites, 34,513 qualified EGD images (Dataset 2) were collected. Five experienced endoscopists labeled EGD images into 26 different sites by following screening protocol 11. To identify the best status of DRL, the researchers used 30 stored EGD videos. To test the clinical performance of the system, a total of 107 stored real EGD videos were used (Dataset 3).

A total of 324 patients with EGD were recruited, of whom 153 patients were randomized in the endoscopic WISENSE-assisted group and 150 in the control group. The accuracy obtained by the system in monitoring blind spots in real EGD videos was 90.40% and the blind-spot rate was significantly lower in the WISENSE group vs control (5.86% vs 22.46%). Additionally, WISENSE increased the inspection time and the completeness of photo documentation.

The WISENSE study has several limitations, such as, first of all, the validation of the performance of their specific algorithm was not made on a large and representative testing data set that included sufficient negative controls before initiating the clinical trials; second, the prolonged inspection time might be a confounding factor for improved lesion detection; third, the study was limited to the Chinese population and Chinese endoscopists (lacks external validation). It is a promising study but the result of an open-labeled single-center study cannot lead to a definite conclusion regarding its ability to improve the quality of upper endoscopy.

The authors presented data on the implementation of the novel AI system ENDOANGEL (previously known as WISENSE) for improving endoscopic visualization within several modalities of EGD [44, 45]: sedated conventional EGD (C-EGD), unsedated C-EGD, and ultrathin transoral endoscopy (U-TOE). ENDOANGEL enables real-time assessment of blind-spot areas using a virtual stomach model and informs the endoscopists on the inspection time. This study was a single-center study that enrolled 437 patients who were initially randomized into one of the three endoscopic modalities. Subsequently, patients in each subgroup were randomized to undergo their EGD with or without ENDOANGEL assistance. A complete endoscopic examination consisted of the visualization of 26 sites. All videos were independently reviewed by five endoscopists. The blind-spot rate was lowest in the sedated C-EGD group. The use of the ENDOANGEL system reduced this rate among all three endoscopic procedures, by 84.77% in the C-EGD, 27.24% in the U-TOE, and 26.45% in the unsedated C-EGD groups. One limit might be the increase in the procedure time. These results suggest a possible benefit of ENDOANGEL in improving the quality of EGD in visualizing mucosa. Therefore, this approach might be used in endoscopic training programs and in assessing the skills of endoscopists.

EGD is the principal modality used in the diagnosis of upper-GI diseases (reflux esophagitis, gastritis, gastroduodenal ulcer, gastric cancer, and others) [46, 47]. Although EGD is a routinely performed procedure, endoscopists require special training and skills to correctly identify these lesions because the endoscopic changes might be rather discrete or resemble other conditions, leading to misdiagnosis [48]. CNN systems have been used to support the diagnosis of GI diseases and reduce the workload on physicians. The first major step for the CAD system is recognizing the anatomical location of the lesion.

A CNN diagnostic system based on the GoogLeNet structure was constructed by Takiyama *et al*. [49] and was trained using a large data set of 27,335 EGD images from 1,750 patients who were grouped into four major anatomical locations (larynx, esophagus, stomach, and duodenum) and three subclassifications for the stomach regions (upper, middle, and lower). The efficacy of the artificial model was evaluated using an independent validation set of 17,081 EGD images from 435 patients. The CNN determined the appropriate anatomical location for 16,632/17,081 EGD images (97%), obtaining high-performance receiver operating characteristics (ROC) curves with area under the curves (AUCs) of 1.00 for recognition of the larynx and esophagus, and 0.99 for the stomach and duodenum. The CNN obtained sensitivity/specificity of 93.9/100% for the larynx, 95.8/99.7% for the esophagus, 98.9/93.0% for the stomach, and 87.0/99.2% for the duodenum. The system achieved AUCs of 0.99 for recognition of the upper, middle, and lower stomach, and sensitivity/specificity values of 96.9/98.5%, 95.9/98.0%, and 96.0/98.8%, respectively. This CAD system displayed good performance in recognition of the anatomical location of endoscopic images, revealing its potential for clinical application in the near future.

### Computer-aided endoscopic navigation system (CAEN)—retargeting gastric biopsies

Conventionally, endoscopists use biopsy for endoscopic follow-up of premalignant gastric lesions in association with tattooing to mark the location of the lesions. Because this is an invasive, sometimes difficult, and inefficient procedure, several studies have emerged that address gastric-biopsy surveillance by means of computer simulation and optical instruments. These research studies included biopsy-relocalization methods based on epipolar geometry [50], the development of a computed-tomography virtual 3D model as a non-invasive biopsy marking system [51], and an online deformation method for pathological-site retargeting [52] or probe-based confocal laser endomicroscopy [53]. However, most of these methods were associated with different difficulties or drawbacks.

A Chinese group of researchers [54] designed a *computer-aided endoscopic navigation (CAEN) system* as non-invasive biopsy procedure, which included a six-degree-of-freedom tracking endoscopic instrument plus a computer-simulated workstation. To visualize the navigation scene, a 3D stomach model was designed based on simultaneous localization and mapping. The tip of the endoscope was used to touch the lesion and the lesion location was subsequently recorded to guide retargeting of the pathologic area during surveillance. This study enrolled 22 participating volunteers and an experienced endoscopist. The experimental data demonstrated that the CAEN procedure needs a shorter time than tattooing and that the system error for the gastric antrum and angulus is <1 cm. Considering that a biopsy forceps opening is ∼6 mm and the tattooing area diameter is ≥1 cm (due to diffusion) [55], this AI system seems appropriate for clinical practice. However, this CAEN system cannot be yet applied to the gastric body with sufficient accuracy and requires optimization.

### Gastric precancerous-lesion detection

#### Gastric precancerous disease network (GPDNet)

The Chinese study by Zhang *et al*. [56] elaborated on CNN classification of 3-class gastric precancerous diseases, namely polyp, erosion, and ulcer, using a model known as the gastric precancerous disease network (GPDNet). After image augmentation, a total of 3,673 gastroscopic images were available, including 1,211 of erosion, 1,218 of polyps, and 1,244 of ulcers. The data set was split into a training set (75%) and a test set (25%). The innovation of the GPDNet consisted of the introduction of fire modules from SqueezeNet to reduce the size and parameters of the system (by ∼10 times) with the purpose of improving the speed of classification. To maintain accuracy with fewer parameters, after training the GPDNet, the authors used iterative reinforced learning (IRL) to fine-tune the parameters, the values of which are close to 0, and considered the modified system as a pretrained model for the next training. IRL seemed to improve the accuracy by ∼9% after six iterations, reaching 88.90%. However, the test set used in this study represented only one-third of the training set, and it was not representative enough. Therefore, the reliability of such results was also uncertain.

Furthermore, the GPDNet was not specifically designed for precancerous disease, such as chronic atrophic gastritis (CAG) or mucosal low-grade dysplasia (LGD)/high-grade dysplasia (ulcerated shape or protusive adenomas). The target was identified based on the macroscopic aspect instead of histology; therefore, the GPDNet was not able to make a distinction between real precancerous lesions and benign erosions/ulcers and fundic-gland polyps.

**Figure 4.**
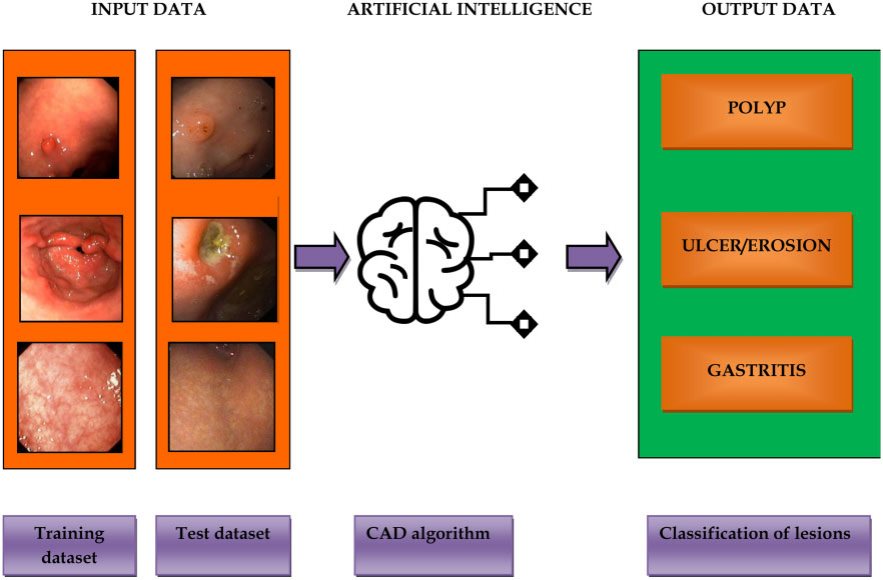
AI algorithms for the classification task of premalignant gastric lesions. Deep-learning (DL) algorithm: the model is trained using a large data set of endoscopic images; subsequently, the classification task performance of the system is validated using an independent test data set.

Figure 4 illustrates the capacity of AI algorithms to perform the classification task of premalignant gastric lesions.

#### Gastric-polyp detection

Although it is known that most gastric polyps are benign, adenomatous polyps must be identified and resected in time because they have the potential for malignant transformation [57]. In this context, it is important to develop automatic gastric-polyp detection to help clinicians to reduce the gastric-polyp miss rate [58].

Initially, most of the AI methods designed to detect and classify digestive polyps used manually designed features such as the elliptical shape [58–60], texture [61], color, location [62], or combinations of these [63]. The methods were time-consuming, associated with a high false-positive rate, and they focused mostly on the detection of colonic polyps.

With the introduction of CNN into endoscopic-lesion classification and detection [56, 64, 65], the accuracy and celerity of detection were improved [66, 67]. Within the MICCAI 2015 Endoscopic Vision Challenge [68], a comparative evaluation was performed by polyp-detection methods in video colonoscopy. The results revealed that none of the presented methods was able to achieve real-time automatic polyp detection. In the next years, the progress of AI tools for polyp detection started to get close to the requirements of the real-life setting. Wang *et al.* [69] developed a DL algorithm for the detection of polyps during real-time colonoscopy by using data from 1,290 patients. For the development data set, colonoscopic images with/without polyps were used. The system was validated on four different data sets: on newly collected colonoscopy images/patients with at least one detected polyp (Dataset A); on a public database of polyp-containing images (Dataset B); on colonoscopy videos with histologically confirmed polyps (Dataset C); and on unaltered full-range colonoscopy videos without polyps (Dataset D). The working group developed an automatic polyp-detection system based on DL with high overall performance in both colonoscopy images/videos, achieving the application requirements of the real-world colonoscopy setting. Therefore, the ideal automatic poly-detection software must use representative data sets and rigorous validation, meaning that validation data sets should be prospectively collected from consecutive patients, with no overlap existing with the training data set; it must have uniform and high overall performance; moreover, it must have low latency, operating with a real-time detection speed.

Billah *et al*. [70] proposed an automatic system that captures the video streams from endoscopic video to support GI polyp detection. The extracted color wavelet (CW) features and CNN features of the video frames are fused to train a linear support vector machine (SVM). Performance assessment on standard public databases revealed that the model outperformed previous methods, reaching an accuracy of 98.65%, sensitivity of 98.79%, and specificity of 98.52% in the identification of polyps.

Zhang *et al*. [10] constructed a CNN based on single shot multibox detector (SSD) architecture known as SSD for gastric polyps (SSD-GPNet). To obtain higher performance and to benefit from the maximal quantity of information from the feature pyramid, the algorithm reused information lost from the pooling layers and joined that extra data to enhance the detection accuracy of the system. The CNN structure merged feature maps of the lower and upper layers to strengthen the relationships between layers. The experiment was performed on the combination of training and test data sets composed of 404 gastric-polyp endoscopic images. The authors did not categorize polyps according to their histology. They divided polyp size into three categories, namely small, medium, and large. More than half of the polyps were small. The system was able to achieve real-time gastric-polyp detection with a speed of 50 frames per second and increased the mean average precision from 88.5% to 90.4%. The authors stated that SSD-GPNet had an improved polyp-detection rate in comparison with the previous version of the AI algorithm, and therefore it improved the polyp-detection rate by increasing especially the detection of small polyps. However, the data set included only a small number of images (404 images of gastric polyps) and the authors did not use the fundamental metrics to assess the performance of AI tools. Instead, the study used performance metrics such as precision and recall, highly affected by the proportion of positive samples in total samples, while the number of true negative results was not stated. Because of the limitations of this study, future research is needed for implementing SSD-GPNet in improving the detection of gastric lesions.

Because measurement of polyp sizes proved to be essential in performing an appropriate *in situ* endoscopic assessment and treatment, 3D endoscopic systems have been designed, including an active stereo-technique, which appears to be promising and accurate, but certain difficulties were encountered in previous research. To overcome these problems, the study group of Furukawa [71] proposed a learning-based algorithm for active stereo using CNN. As such, two independent networks (U-Nets) were developed and trained for tasks such as line detection and code-based segmentation. In addition, to reconstruct a unified shape from multiple scans for an endoscopic device, an extended bundle-adjustment technique was elaborated that estimated the 3D shapes and the calibration parameters at the same time. The performance of the proposed methodology vs previous research [72] was evaluated experimentally using human resected tumor samples, a liver phantom, and a pig stomach. The CNN training data set was obtained from 47 real endoscopic images through a projector–camera system inserted into the endoscope channel. To evaluate the pattern-feature-extraction capacity of the system for endoscopic aspects, resected cancer specimens were measured. The proposed method showed an accurate grid/code-detection capacity and 3D reconstruction of large regions, and therefore it needs further validation using *in vivo* experiments.

#### *Diagnosis of* H. pylori *infection*

*Helicobacter pylori* infection plays a crucial role in the pathogenesis of gastric cancer by inducing a multistep carcinogenic process, including atrophic gastritis, intestinal metaplasia, and eventually carcinoma development [73, 74]. *Helicobacter pylori* infection is associated by an increased risk of gastric neoplasm and, moreover, *H. pylori* eradication leads to a decrease in gastric-cancer incidence. Therefore, the International Agency for Research on Cancer defined *H. pylori* as a class I carcinogen [75–77]. EGD is frequently performed for the screening of gastric cancer and for the detection of other *H. pylori*-related diseases. Endoscopic assessment also appears helpful in diagnosing *H. pylori* infection because findings such as diffuse erythema, edema, atrophy, prominent folds, and nodularity are typical for *H. pylori* infection, whereas a regular architecture of collecting venules and the presence of fundic-gland polyps are characteristic of the absence of infection. Furthermore, the map-redness aspect of gastric mucosa is mostly observed in eradicated patients [78]. However, endoscopic evaluation is time-consuming and subjective, and depends on the experience of the endoscopist.

Initially, in 2004, Huang *et al*. [79] investigated the presence of *H. pylori* infection based on refined feature extraction by means of a neural network using endoscopic images and their related gastric pathologic aspects. This model was trained/analysed with 84 image parameters collected from 30 patients and was associated with a sensitivity and specificity of 85.4% (35/41) and 90.9% (30/33) for *H. pylori* infection and an accuracy for the identification of gastric atrophy, intestinal metaplasia, and severity of inflammation that exceeded 80% (83.8%, 89.2%, and 83.8% respectively). The gold standard used was the histological features. Additionally, Itoh *et al*. [80] developed a CNN model to predict *H. pylori* infections using a total of 596 endoscopic images (after data augmentation of 179 gastroscopic images obtained from 139 patients, the presence or absence of HP was confirmed by detecting the serum HP IgG antibodies in those patients), which showed promising results with a sensitivity and a specificity of 86.7% and an AUC of 0.956.

In 2017, Shichijo *et al*. [81] designed a CAD system to improve the endoscopic diagnosis of *H. pylori* infection. First, that group constructed a 22-layer CNN that was pretrained and fine-tuned using a data set of 32,208 endoscopic images from either *H. pylori*-positive or -negative patients (735 and 1,015 patients, respectively). All of the patients included were assessed for *H. pylori* using at least one non-invasive test and were considered *H. pylori*-infected if any of these assays became positive. Subsequently, a secondary CNN was trained using endoscopic images classified according to eight different anatomical locations in the stomach. A separate test data set including 11,481 images from 397 patients, of whom 72 were *H. pylori*-positive and 325 were negative, was independently assessed by the AI system and by 23 endoscopists. The first CNN achieved a sensitivity of 81.9%, a specificity of 83.4%, and an accuracy of 83.1% for the detection of *H. pylori* infection, requiring 198 s for analysis of all images. The secondary system obtained a sensitivity of 88.9%, specificity of 87.4%, and an accuracy of 87.7%, requiring 194 s for data analysis. The secondary CNN obtained a significantly higher accuracy than endoscopists for the detection of infection in a notably short interval of time (only several minutes).

Because this study included only *H. pylori*-positive and -negative patients and excluded patients after *H. pylori* eradication, a situation was demonstrated to be still associated with moderate risk of gastric-cancer development [82, 83]; the group constructed another CAD to evaluate the *H. pylori* status (2019) [9] and pretrained and fine-tuned a CNN using a data set of 98,564 endoscopic images from 5,236 patients, of whom 742 were *H. pylori*-positive, 3,649 were negative, and 845 were eradicated. The performance of the system was evaluated using a separate test data set, including 23,699 images from 847 patients, of whom 70 were *H. pylori*-positive, 493 were negative, and 284 were eradicated. All patients were evaluated for *H. pylori* infection by at least one non-invasive test and considered infected if they tested positive on any of these assays. The trained CNN estimated the probability index for *H. pylori*-infection status per image and subsequently selected the final “diagnosis” (highest probability) of the infection condition. Among the 23,699 test images, 418 were classified as *H. pylori*-positive, 23,034 as negative, and 247 as eradicated. After the artificial redefinition of *H. pylori*-negative probability due to the large number of such findings, the system achieved an accuracy of 80% (465/582) for *H. pylori*-negative cases, 84% (147/174) for eradicated infections, and 48% (44/91) for positive cases—a performance comparable to that of experienced endoscopists. The system required a total of 261 s to diagnose all of the images from the test data set.

The use of CAD during the endoscopic procedure might offer quick risk stratification of patients for the development of gastric cancer according to *H. pylori* status. This advantage is of maximum importance, especially in countries with a high incidence of gastric cancer. In Japan, for example, *H. pylori* eradication in patients with gastritis (indeed, all infected patients) has been covered by national health insurance since 2013 and, furthermore, endoscopic mass screening for gastric neoplasm was initiated in 2016. These programs led to an overwhelming workload for endoscopists, which might be relieved with the aid of a CNN diagnostic algorithm.

The *image-enhanced endoscopy* (*IEE) device* was shown to improve non-magnifying endoscopic detection of *H. pylori* gastritis [84]. In this direction, Nakashima *et al*. [29] structured a prospective single-center pilot study with the aim of designing an AI diagnosis algorithm that predicts *H. pylori*-infection status using this modern endoscopic technique. Training of the AI system used a data set including a total of 222 subjects (of whom 105 were *H. pylori*-positive) who underwent EGD, with subsequent selection of three still images of the lesser gastric curvature/each case using WLE, blue-laser imaging (BLI)-bright, and LCI, respectively. A total of 1,944 endoscopic images were prepared for the AI training, 648 for each group (WLE, BLI-bright, and LCI). Subsequently, 180 images from the test data set (60 for each endoscopic modality) were classified. A serologic test for *H. pylori* IgG antibody titer was considered as the gold standard for infection status. This study used a GoogLeNet 22-layer CNN as a pretrained and fine-tuned model that acquired image-recognition capability as an endoscopic CAD system.

The AUC obtained was only 0.66 for white light imaging but was excellent for BLI-bright and LCI modes, with values of 0.96 and 0.95, respectively. Moreover, the sensitivity of the artificial CNN for BLI-bright and LCI was 96.7%. Thus, CAD in association with IEE is expected to become a useful endoscopic diagnostic tool in the near future.

*Linked color imaging (LCI)* was demonstrated to detect slight differences in mucosal color, such as those encountered in the case of gastritis, metaplasia, or atrophy [84, 85]. A Japanese research study [86] proposed a universally interpretable AI multistage algorithm for the endoscopic diagnosis of *H. pylori* infection based on LCI. The training data set included 128 endoscopic images (4 images each from 32 cases). Patients were assessed for *H. pylori* infection using more than two different tests. Based on a slight difference in redness, gastric images were categorized into two patterns (high-hue and low-hue images) for training of SVM classifiers to automatically detect *H. pylori* infection. To investigate the performance of the learning machine, it was used on a test data set including 525 LCI endoscopic images from 105 patients (5 images from different regions per case), of whom 42 were *H. pylori*-positive, 46 were post-eradication, and 17 were negative. The AI system obtained an accuracy of 87.6% (92/105), sensitivity of 90.5% (38/42), specificity of 85.7% (54/63), and positive predictive value of 80.9% (38/47), and negative predictive value of 93.1% (54/58). These performance scores were higher than those of inexperienced endoscopists and quite similar to those of expert endoscopists. However, the study currently has selected drawbacks, mostly related to the ability to diagnose post-eradication patients.

Discrete lesions, containing subtle changes in color, morphology, or texture, might generate similar detection difficulties both for AI and for doctors. AI may be able to achieve a superior diagnostic performance by summarizing the variation rules of the features characterizing the pathologic superficial epithelium, but this method is still an indirect approach when compared with pathological assessment and also may have its in-nature limitations.

#### CAG detection

CAG represents a major stage in the carcinogenic process of gastric cancer [87] and its extent is correlated with the risk of cancer development [88–90]. Eradication of *H. pylori* infection can significantly improve atrophy in these patients, with a subsequent reduction in the risk of gastric neoplasm. For this reason, it is essential to detect CAG to prevent the development of gastric cancer [91]. As such, specific guidelines exist for the endoscopic assessment of precancerous conditions using standardized biopsy protocols [92]. However, multiple biopsies increase mucosal trauma and risk of bleeding, and they are also costly and time-consuming. Recently, although advanced endoscopy techniques such as magnifying endoscopy (ME)-CE and confocal laser microscopy improved the diagnosis accuracy of CAG [93], they proved useful tools only in the hands of experienced endoscopists. Moreover, these advanced techniques are not widely available and require expensive devices. Because the morphological characteristics of gastric atrophy are discrete and difficult to detect, CAD of atrophic gastritis emerged as a necessity. Therefore, DL technology has begun to be used in diagnosing pathological images of CAG [94]. Lahner *et al*. (2005) constructed an ANN and linear destructive analysis system based on clinical and biological data to support the diagnosis of CAG without endoscopy—an artificial model associated with an accuracy rate of 100% [95]. Because patients with atrophic gastritis might present non-specific symptoms and gastroscopy with biopsy still remains the main diagnostic modality, it is mandatory to develop DL techniques that can assist in the endoscopic screening of CAG.

The research group of Guimarães [96] structured a CAD approach for the diagnosis of CAG and the approach was trained using real-world endoscopic images originating from the proximal stomach. The training data set consisted of 200 WLE images from patients with and without histology-proven CAG (100 from each group) that were exported using the digital imaging and communications in medicine (DICOM) format. Subsequently, data were augmented using the artificial techniques of rotation, mirroring, and scaling. An independent test data set of 70 images (30 with CAG and 40 without) was used in evaluation by six endoscopists with different degrees of expertise. The CAD system used a pretrained fine-tuned CNN. Initially, the best architecture was assessed using pretrained models on ImageNet and a 10-fold cross-validation was performed on the training set. For each cross-validation, data were split into training (80%), tuning (10%), and testing (10%) sets. The test data set was classified according to the best-performing combination of the hyperparameters. The model obtained a diagnostic accuracy of 93% and an AUC of 0.98, showing significantly better performance than endoscopists from the tertiary referral center. Histopathology was used as the gold standard.

The study by Zhang *et al*. [97] (2020) designed a CNN model to improve the diagnostic accuracy of CAG based on 5,470 images of gastric antrums (3,042 images of atrophic gastritis and 2,428 of non-atrophic gastritis) from 1,699 patients who were labeled with their pathological features. Endoscopic images were randomly assigned to the training set (70%) and testing set (30%). Subsequently, the training data set underwent 5-fold cross-validation and the diagnoses of the artificial model were also compared with those of three endoscopist experts. The CNN model obtained a diagnostic accuracy, sensitivity, and specificity of 0.942, 0.945, and 0.940, respectively—performance scores that were better than those of the experts. In addition, the detection rates according to the severity of the lesions were 93% for mild atrophy, 95% for moderate atrophy, and 99% for severe atrophic gastritis.

These promising results highlight the usefulness of the CNN model in diagnosing CAG, by achieving a higher accuracy and by reducing the burden on endoscopists.

### Gastric-cancer detection

Stomach cancer remains a major global health problem and is responsible for >1,000,000 new cases and 783,000 deaths worldwide (2018). Stomach cancer is the fifth most frequently diagnosed cancer globally and the third leading cause of cancer death [98]. Large geographic variation occurs in the incidence of gastric cancer, with notably high rates in Eastern Asia, in contrast to other regions such as Northern America and Northern Europe with lower incidences. *Helicobacter pylori* is considered the main risk factor and is responsible for almost 90% of non-cardia gastric-cancer cases [99, 100]*.* The incidence of non-cardia gastric tumors has been steadily declining over the past decades, mostly due to prevention measures, including a decrease in *H. pylori* prevalence and improvements in the preservation of foods. The characteristics of gastric cardia cancers more closely resemble those of esophageal adenocarcinoma, obesity, and gastroesophageal reflux disease, with Barrett’s esophagus representing important risk factors. With respect to the incidence of these specific cancers, an increasing trend has been noted, especially in developed countries.

Early diagnosis and treatment of gastric cancer determine an improvement in the 5-year survival rates to 96% compared with the high mortality and poor prognosis associated with advanced tumors [101]. Therefore, establishing a diagnosis of gastric cancer at an early stage represents a major step forward in the management of this tumor.

For the classification of gastric cancer, Sun *et al*. [102] used VGGNet, IRNV2, and the R-FCN algorithm to classify benign vs malignant gastric ulcers. The best accuracy of 86.6% was obtained using the R-FCN network—a result that exceeded the performance of doctors.

It is important to emphasize the difference between the object-detection task and the classification task. Detection means the ability of AI to recognize a ROI, such a dysplastic/neoplastic area (which can be histologically confirmed subsequently using tissue biopsy), while classification means the ability of AI to categorize the identified lesions into different classes (e.g. neoplastic vs non-neoplastic). This signifies that, for a lesion to be categorized (classification), it definitely should be first of all visualized (detection). Nowadays, optical biopsy has gained increasing accuracy, but still tissue biopsy represents the gold standard for diagnosing gastric lesions.

#### CAD using CE

Starting from the consideration that a hybrid AI approach could be suitable for the advanced assessment of various descriptors to represent the characteristics of gastric images, Ali *et al*. [103] proposed a new hybrid feature-extraction method known as the Gabor-based gray-level co-occurrence matrix (G2LCM) used in CADe of CE abnormal frames. The Ali group compared the performance scores of multiple types of classifiers by training them initially on features obtained from existing texture-extraction methods and subsequently on the G2LCM matrix.

The study used a publicly available database consisting of 176 CE images of multiple patients with normal aspect/abnormal tumor lesions and associated with two sets—one for training and the other for testing the methods. These images were also classified according to color histograms [104]. The data set included three groups: 56 (31.8%) images of normal mucosa—group I (normal class) and 120 (68.2%) images with lesions of metaplasia and dysplasia—groups II and III (abnormal class). AI algorithm performance was compared to the assessment of two expert endoscopists.

Using an SVM classifier and G2LCM texture features, abnormal images were differentiated from normal frames with a sensitivity of 91% (110/121), specificity of 82% (45/55), accuracy of 88% (155/176), and an AUC of 0.91. These results demonstrate that the proposed system can be used to assist gastroenterologists in gastric neoplastic screening.

Ogawa *et al*. [105] performed an objective assessment of the utility of different chromoendoscopy techniques for EGC detection by means of a ML algorithm (SVM) that uses data of color differences. A total of 54 still endoscopic images from 18 histopathologically confirmed EGC lesions were examined and endoscopic images from WLE, indigo carmine (Indigo), and acetic acid-indigo carmine chromoendoscopy (AIM) were prepared for the CADe system. A border distinguishing between cancerous/non-cancerous areas on the endoscopic images was delineated from post-treatment pathological findings. Each pixel was considered as a sample and was represented as a 3D vector using RGB values. The study evaluated the Mahalanobis distance as indicative of color differences between cancerous vs non-cancerous areas. Subsequently, a diagnostic test using an SVM was performed for each image. The model was trained using 100 samples per class and evaluated which area each of the 1,900 samples per class originated from.

The obtained means for the Mahalanobis distances did not differ significantly for the three modalities. The diagnostic ability per endoscopy technique was assessed using the F1 measure. The objective assessment using SVM was helpful for confirming the superiority of AIM images to detect EGC.

#### CAD using flexible spectral imaging color enhancement (FICE)

Miyaki *et al*. [106] reported software designed to automatically distinguish cancerous/non-cancerous areas using a bag-of-features framework with densely sampled scale-invariant feature-transform descriptors to magnify ***FICE*** (Fujifilm Corp., Tokyo, Japan) images. Although based on small data sets, the study was well designed and collected meaningful negative samples, which were similar in morphology and color, as control. While it is a relatively easy task for an AI tool to be sensitive to detect early cancer, it may be extremely difficult for the system to be specific to differentiate benign from malignant lesions with similar characteristics. The CAD system was validated using 46 intramucosal gastric cancers and reached an accuracy of 85.9% (79/92), sensitivity of 84.8% (39/46), and specificity of 87% (40/46) for cancer diagnosis; also, it achieved a positive predictive value of 86.7% (39/45) and negative predictive value of 85.1% (40/47).

#### CAD using hyperspectral imaging

*Hyperspectral imaging (HSI)* is an emerging domain that combines ML with spectroscopy [107] and acquires 2D images containing spectral information for each pixel, thus supplying better contrast than white-light imaging. Cancer detection represents one of the principal applications of HSI in the medical field [108] and the method demonstrated efficacy in identifying different types of tumors [109–111]. Previous research on the colon or esophagus using HSI was performed only on *ex vivo* specimens [112] or histopathological fragments [113].

The research group of Hohmann [114] proposed (2017) multispectral imaging (MSI) as an initial step towards hyperspectral video endoscopy (HSVE). For this reason, a standard endoscopy system was modified to perform *in vivo* multispectral imaging of the upper digestive tract, associated with the automatic classification of normal/cancerous areas using SVM. The pilot study was conducted on 14 gastric-cancer patients and different classifiers were compared for data classification based on a leave-one-out strategy. For system training, the tumor limits were outlined by expert-labeling and tumoral lesions were histologically confirmed. The technology achieved a sensitivity of 63% and specificity of 64% (for the best classifier). Although this study faced selected difficulties due to the current level of the technology, it might be possible to transfer the results to hyperspectral imaging and solve certain limits of the methodology in the future.

#### CAD using BLI

*BLI combined with ME* was demonstrated to improve diagnostic accuracy for EGC and precancerous lesions [115]. The group of Miyaki *et al*. [116] constructed an SVM-based system to quantitatively identify gastric cancer on images obtained by ME-BLI. The system was evaluated on 100 consecutive EGC in 95 patients and produced a data set from 100 images of EGC, 40 ﬂat/slightly depressed, small, reddened mucosa (benign lesions) and the surrounding tissues. The authors attempted to quantitatively distinguish the lesions. The SVM output value for cancerous lesions was significantly greater than that for reddened lesions/surrounding tissue, and thus it allows the quantitative diagnosis of gastric lesions.

#### CAD using NBI

The endoscopic technique of magnifying endoscopy with narrow-band imaging (ME-NBI) has been applied to enhance EGC detection by describing the microvascular pattern and microsurface architecture of gastric mucosal lesions [117]. However, in the hands of non-experts, this approach acquired only modest diagnostic efficacy in differentiating cancerous from non-cancerous gastric lesions.

To boost the diagnostic accuracy, Kanesaka *et al*. [24] created a CAD system using an SVM to enhance the efficiency of ME-NBI in distinguishing EGC and to delineate the border between cancerous and non-cancerous areas. The performance of the system was compared to the expert endoscopists’ region delineation. A total of 126 magnifying NBI images were used as training material. In this pilot study, the authors trained the model using 61 ME-NBI images of EGC and tested it on 20 ME-NBI images of non-cancerous areas. The results highlighted a remarkable diagnostic performance (accuracy of 96.3%, positive predictive value of 98.3%, sensitivity of 96.7%, and specificity of 95%) and performance of area concordance (accuracy of 73.8%, sensitivity of 65.5%, and specificity of 80.8%).

A Chinese group [118] analysed the performance of EGC detection using transfer learning with CNN on magnifying NBI endoscopic images. The VGG16, Inception V3, and InceptionResNetV2 algorithms were selected to accomplish the image-classification task. The coarse data set of M-NBI images was used in training/testing a total of 1,438 EGC and 1,630 normal endoscopic images, out of which a fine data set was further selected. Experiments showed that DL using CNN performs better than traditional handcrafted methods. The best performance scores were obtained by fine-tuning Inception V3, with accuracy, sensitivity, and specificity of 0.985, 0.981, and 0.989, respectively.

Li *et al*. [119] developed a new CNN system to classify gastric mucosal lesions detected by ME-NBI and constructed a CNN model (Inception-v3) trained using 386 endoscopic images of non-cancerous lesions and 1,702 images of EGC. The diagnostic efficacies of both the ML system and endoscopists were tested on a data set of 341 endoscopic images containing 171 non-cancerous lesions and 170 EGC. The histopathologic result was considered the gold standard. The CNN system achieved sensitivity, specificity, and accuracy of 91.18% (155/170), 90.64% (155/171), and 90.91% (310/341), respectively, in the diagnosis of EGC. Although comparable specificity and accuracy of diagnosis were noted between CNN and experts, the diagnostic sensitivity of CNN was significantly better. Furthermore, the diagnostic performance scores were significantly higher than those of the non-experts. Therefore, the use of this CAD system could be notably helpful in gastric-cancer screening.

Hirasawa *et al*. [27] constructed a CNN system based on SSD architecture to detect early and advanced gastric cancer. The CNN was trained by a data set including 13,584 non-magnified WLE/indigo carmine CE/NBI endoscopic images for 2,639 histologically proven gastric-cancer lesions. The diagnostic accuracy was evaluated using an independent test data set composed of 2,296 WLE images of 77 gastric cancers gathered from 69 consecutive patients.

The CNN analysed all of the test images in <1 minute. The proposed system correctly diagnosed 71/77 gastric-cancer lesions, achieving an overall sensitivity of 92.2%, whereas 161 non-tumoral lesions were misdiagnosed as gastric cancer, leading to a positive predictive value of 30.6% (71/232). The system diagnosed 70/71 lesions (98.6%) that were >6 mm in diameter and all invasive tumors. All of the undetected cancers were intramucosal neoplasms of a superficially depressed and differentiated type that might be confounded with gastritis, even by expert endoscopists. However, nearly half of the false-positive cases consisted of gastritis lesions expressing marked changes in mucosal coloration or surface architecture that might mimic neoplastic alterations.

In the study by Hirasawa *et al*., many endoscopic images of gastritis were misdiagnosed as EGC, and therefore it was considered that the AI achieved a quite low probability of differentiation. Thus, to further explore the possibility that a DL system can improve EGC diagnosis in clinical practice, Horiuchi *et al*. [21] used magnified endoscopic images. The CNN system composed of 22 layers was pretrained using 1,492 EGC/1078 gastritis ME-NBI images. For assessment of the diagnostic performance of the CNN, an independent test data set including 151 EGC/107 gastritis ME-NBI images was used. The histopathologic result was considered the gold standard. The diagnostic ability achieved by the system was described as follows: accuracy 85.3%, sensitivity 95.4%, specificity 71.0%, positive predictive value 82.3%, and negative predictive value 91.7%. Additionally, 7/151 EGC were misdiagnosed as gastritis (false negative), whereas 31/107 gastritis images were classified as EGC (false positive). The main causes of misdiagnosis were represented by localized atrophy/atrophy of the fundic gland and intestinal metaplasia. The CNN produced a high AUC of 85.2%. These promising results demonstrate that the CAD system using ME-NBI stored endoscopic images is capable of rapid differentiation between EGC and gastritis with a high sensitivity and negative predictive value, and is helpful in the daily clinical routine.

#### CAD using video endoscopic images

To implement this methodology in the real-time detection of gastric cancer in the screening EGD program, a pilot study was initiated to evaluate the performance of a CNN algorithm when applied to video endoscopic images [120]. To test the accuracy of detection, video images of 68 endoscopic submucosal dissections performed in 62 patients using EGC were assessed. CNN obtained an accuracy of detection similar to that of still images by correctly diagnosing 64/68 tumors (94.1%). The median time needed for detection was 1 s per lesion. Similarly to the previous work, in the four missed cases, the neoplastic changes were difficult to distinguish from background gastritis. Although the sensitivity and latency of this study seem good, the specificity of the AI tool was not analysed. Therefore, it is unclear whether the high sensitivity of this AI system is based on a compromise of false positives.

The proposed system has the ability to conduct rapid analysis of a large number of stored/video endoscopic images and achieves good diagnostic accuracy for gastric cancer. Thus, this approach might be implemented in clinical practice to relieve the burden on endoscopists, especially when used as a support tool for the gastric-cancer screening program, for telemedicine in rural areas and developing countries, or for second-opinion purposes. In this manner, the standard for EGC detection is expected to increase in the future.

### Invasion-depth detection of gastric cancer

Correct assessment of the neoplastic-invasion depth is mandatory for proper selection of patients with EGC for endoscopic resection. For this purpose, in 2012, Kubota *et al*. [121] were the first to evaluate a CAD system used to identify the depth of the wall invasion of a gastric neoplasm using endoscopic images. These researchers collected 902 endoscopic images and created a backpropagation model defined by 10-time cross-validation, with an achieved diagnostic accuracy of 77.2% (346/448) for T1 (68.9% (157/228) T1a/63.6% (140/220) T1b), 49.1% (52/106) for T2, 51.0% (76/149) for T3, and 55.3% (110/199) for T4 staging.

Zhu *et al*. [22] developed a CAD system using the transfer learning methodology based on a pretrained CNN architecture (ResNet50). The AI system used a total of 790 endoscopic gastric-cancer images as a development data set, including 632 images for the training data set and 158 for the validation data set. After data augmentation, the number of images for these data sets increased to 5,056 and 1,264 images, respectively. To evaluate the classification accuracy of the system, another 203 images were used as a test data set. The results were compared with those of 17 endoscopists of varying experience. The CNN-CAD system achieved an AUC of 0.94. At a threshold value of 0.5, the model obtained a sensitivity of 76.47% and a specificity of 95.56%. The overall accuracy was 89.16%, whereas the positive and negative predictive values were 89.66% and 88.97%. The accuracy and specificity achieved by the system in estimating the invasion depth of gastric cancer were significantly higher than those obtained by the endoscopists. This system was able to distinguish between EGC with superficial invasion from cancers with deeper infiltration of submucosa, thus reducing overestimation of the invasion depth and the number of surgical resections, leading to an improved outcome and quality of life for those patients. Although promising, it was a single-center study, based on a small sample size.

### Novel AI endoscopic devices and data-set platforms for upper-digestive-cancer detection

Currently, AI software is incorporated in devices that are routinely used. The first commercial AI system included in endoscopic techniques, known as EndoBRAIN, was developed in collaboration with expert Japanese endoscopists and was launched in March 2019 (Olympus) [122, 123]. This system allows the differentiation of neoplastic vs non-neoplastic polyps during real-time colonoscopy [124]. Huiyan Luo and colleagues [125] analysed the application of an AI system in WLE upper-GI endoscopy in a real-life setting. This study was the first to incorporate a notably large number of 1,036,496 endoscopy images from 84,424 individuals across six hospitals of different tiers in China to train and validate a GI AI model for the diagnosis of upper-GI cancer, known as the Gastrointestinal Artificial Intelligence Diagnostic System (GRAIDS). This system was based on the retrospective collection of endoscopy images and was subjected to both internal and external prospective validation. The CAD obtained a diagnostic accuracy of 0.955 in the internal validation data set, 0.927 in the prospective set, and between 0.915 and 0.977 in the five external validation sets. The diagnostic sensitivity obtained by GRAIDS was similar to that of the expert endoscopist (0.942 vs 0.945). The positive predictive value of GRAIDS was lower vs expert endoscopists. Converting still endoscopic frames to video analysis was overcome by GRAIDS, which is capable of processing a minimum of 25 images/second with a latency of <40 ms. Based on the high accuracy of GRAIDS in diagnosing upper-GI cancers, the study group constructed a cloud-based multi-center AI platform to supply real-time assistance during gastroscopy and a freely accessible website for telemedical assistance and second opinions in difficult cases.

Table 1 summarizes the previously described studies regarding AI algorithms in upper-endoscopy-quality monitoring and gastric lesion assessment.

**Table 1.** Current studies applying AI in the assessment of gastric lesions

Ref.	Published year	Aim of study	Design of study	Type of AI (AI classifier)	AI validation methods	Number of subjects								
						Training data set			Test data set			Performance		
						No. cases (negative/positive)	No. images (negative/positive)	Endoscopic procedure	No. cases (negative/positive)	No. images (negative/ positive)	Endoscopic procedure	Accuracy %	Sensitivity/ specificity %	AUC
Wu L *et al.* [30] ChiCTR 1800014809	2019	Real-time quality- improving system for EGD: WISENSE	P	Deep CNN; DRL	10-fold CV	>3,000 pts (Dataset 1)	12,220 *in vitro*, 25,222 *in vivo*, 16,760 EGD images (Dataset 1); 34,513 qualified EGD images (Dataset 2) +30 stored EGD videos (for DRL)	EGD images + videos	150 pts in the control group/153 pts in the endoscopic WISENSE-assisted group	107 stored real EGD videos (Dataset 3)	Video endoscopy	90.40	87.57/95.02 (video EGD)	–
Chen D *et al.* [44] ChiCTR 1900020920	2020	Improving endoscopic visualization within several EGD modalities	P	Deep CNN; DRL	CV	437 pts randomized to 1 of 3 endoscopic modalities → randomized to have their EGD ± ENDOANGEL assistance					Video endoscopy	ENDOANGEL ↓ the blind-spotrate: C-EGD 3.42%; U-TOE 21.77%; unsedated C-EGD 31.23%		
Takiyama H *et al.* [49]	2018	Recognizing the anatomical location of the lesion	R	Deep CNN (GoogLeNet architecture), backpropagation	Fine-tuned using Adam (stochastic optimization)	1,750 pts	27,335 EGD images	WLE	435 pts	17,081 EGD images	WLE	97	93.9/100 larynx; 95.8/99.7 esophagus; 98.9/93.0 stomach; 87.0/99.2 duodenum	1.00 larynx, esophagus; 0.99 stomach, duodenum
Zhang X *et al.* [56]	2017	Gastric precancerous diseases classification	P	CNN= GPDNet – fire modules from SqueezeNet	IRL—6 iterations	– 3,673 gastroscopic images: 1,211 of erosion, 1,218 of polyps, 1,244 of ulcer					WLE images	88.90		–
Billah M *et al.* [70]	2017	Automatic gastrointestinal polyp detection	R	Extracted CW/CNN features (video frames)—fused to train a linear SVM		– >14,000 images (2/3 non-polyp, 1/3 polyp) → 144 CW and 4,096 CNN features/image, fused together					>100 standard EGD videos	98.65	98.79/98.52	–
Zhang X *et al.* [10]	2019	Automatic real-time polyp detection	P	CNN based on SSD-DSSD: SSD-GPNet (VGG16)	Reuse information abandoned by Max–Pooling layers	215 pts/404 gastric polyps (training+ test)	708 polyp images	EGD images	+ 72 pts/182 polyps (Dataset 2)	50 polyp images + 171 polyp images (Dataset 2)	EGD images	mAP 90.4		–
Huang CR *et al.* [79]	2004	Diagnosis of *H. pylori* infection and associated gastric lesions	P	Nonlinear conjugate gradient algorithm for supervised training of a multilayer backpropagation neural network (RFSNN)		30 dyspeptic pts (15 H.p +, 15 H.p –)	84 image parameters/patient	WLE EGD	74 dyspeptic pts (33 H.p –, 41 H.p +)	84 image parameters/patient	WLE EGD		85.4/90.9	–
Itoh T *et al.* [80]	2018	Diagnosis of *H. pylori* infection by learning endoscopic images	P	GoogLeNet deep CNN	Stochastic gradient descent	139 cases (total data set)	149 images → data-augmentation 596 images	WLE EGD	30 (15 H.p –/15 H.p +) cases	30 (15 H.p –/15 H.p +) images	WLE EGD	–	86.7/86.7	0.956
Shichijo S *et al.* [81]	2017	Diagnosis of *H. pylori* infection	R	Deep CNN (22 layers) trained using backpropagation	Caffe deep-learning framework	1,750 (1,015 H.p –/735 H.p +) pts	CNN1: 32,208 images; CNN2: images classified according to 8 different gastric locations	WLE EGD	397 (325 H.p –/72 H.p +) pts	11,481 images	WLE EGD	83.1 (CNN1); 87.7 (CNN2)	81.9/83.4 (CNN1); 88.9/87.4(CNN2)	–
Shichijo S *et al.* [9]	2019	Diagnosis of *H. pylori* gastritis on the basis of endoscopic images	R	Deep CNN GoogLeNet architecture trained using backpropagation	Caffe deep-learning framework, fine-tuned by using stochastic optimization	5,236 pts : 742 H.p +, 3,649 –, 845 eradicated	98,564 EGD images	WLE EGD	847 pts: 70 H.p +, 493 –, 284 eradicated	23,699 images	WLE EGD	80 for H.p-cases, 84% for eradicated infections, 48% for + cases		–
Nakashima H *et al.* [29]	2018	AI diagnosing algorithm that predicts *H. pylori* infection status using IEE system	P, pilot	Deep CNN GoogLeNet 22 layers	framework, Caffe	162 subjects (87 H.p –/75 H.p +)	1944 images (648 for each EGD modality)	WLE, BLI-bright, and LCI EGD	60 subjects (30 H.p –/30 H.p +)	180 images (60 for each EGD modality)	WLE, BLI-bright and LCI EGD	–	96.7 (BLI-bright and LCI)	0.66 (WLE); 0.96 (BLI-bright) 0.95 (LCI)
Yasuda T *et al.* [86]	2020	Automatic diagnosis system with LCI for diagnosis of *H. pylori* infection	R	SVM		32 cases (18 H.p –, 14 H.p +)	128 images (4 EGD images/case)	LCI EGD	105 pts (17 H.p –, 42 H.p +, 46 post-eradication)	525 images (5 images from different regions/case)	LCI EGD	87.6	90.4/85.7	–
Guimarães P *et al.* [96]	2020	Diagnosis of atrophic gastritis	P	CNN (pretrained on ImageNet)	10-fold stratified CV	101 pts (64/37)	200 real-world images (100/100)	WLE EGD	35 pts (22/13)	70 images (40/30)	WLE EGD	93	–	0.98
Zhang Y *et al.* [97]	2020	Diagnosis of chronic atrophic gastritis	R	CNN (DendeNet)	Five-fold CV	– 1699 pts – 5,470 antrum images (2,428 non-atrophic gastritis/3,042 atrophic gastritis)					WLE i-SCAN EGD	94.2	94.5/94	–
Ali H *et al.* [103]	2018	Computer-assisted detection of CE abnormal gastric frames	R	Hybrid feature-extraction method: G2LCM; SVM	10-fold CV	– 176 frames of multiple pts with normal aspect/abnormal tumor lesions → 3 groups: 56 of normal mucosa—group I (normal class); 120 images with metaplasia/dysplasia—groups II/III (abnormal class)					CE EGD	87	91/82	0.91
Ogawa R *et al.* [105]	2018	EGC detection	R	SVM		18 EGC pts/54 EGD still images (database)	100 samples from the non-cc/100 cc areas	WLE, CE, AIM EGD		3,800 samples (1,900 samples from the non-cc/1,900 cc areas)	WLE, CE, AIM EGD	Means of F1 measures: 0.636 WLE, 0.618 CE, 0.687 AIM		
Miyaki R *et al.* [116]	2015	Quantitative diagnosis of EGC using laser-based endoscopy	P	Densely sampled SIFT descriptors in a bag-of-features framework; SVM	VS classification system		587 cutout images of EGC, 503 cutout images of surrounding tissue	ME-BLI EGD	95 consecutive pts	100 images of cc/100 of surrounding tissues; 40 of reddened lesions	ME-BLI EGD	SVM output value: 0.846 ± 0.220 for cc lesions, 0.381 ± 0.349 for reddened lesions, and 0.219 ± 0.277 for surrounding tissue (SVM output value for cc was significantly greater vs reddened lesions/surrounding tissue)		
Hohmann M *et al.* [114]	2017	*In vivo* diagnostics of gastric pre-cancer and cancer using MSVE	P, pilot	SVM with linear and Gaussian Kernel, Ada-Boost, RobustBoost, Random-Forest-walk	Leave-one-out strategy	– 14 GC pts → multiple images of the suspected area					MSVE EGD	64 (for the best classifier RobustBoost)	63/64	–
Kanesaka T *et al.* [24]	2018	Diagnosis of EGC using magnifying NBI images	R, pilot	GLCM features, SVM			126 images (60 non-cc/66 EGC)	ME-NBI EGD		81 images (20 non-cc/61 EGC)	ME-NBI EGD	96.3	96.7/95	–
Liu X *et al.* [118]	2018	Diagnosis of EGC using magnifying NBI images	R	Deep CNN (VGG16, InceptionV3, InceptionResNetV2)	transfer learning by fine-tuning	– Coarse data set (training and testing): 1,630 normal, 1,438 EGC images – Fine data set: 1,120 images (562 normal, 558 EGC)					ME-NBI EGD	98.5 (fine-tuning InceptionV3)	98.1/98.9	–
Li L *et al.* [119]	2020	Diagnosis of EGC using magnifying NBI images	R/P	CNN model (Inception-v3)	Keras deep-learning framework	386 images of non-cc, 1,702 of EGC lesions	↑sample size by Image-processing methods → total of 10,000/10,000 images	ME-NBI EGD	171 non-cc/170 EGC pts	341 images (171 non-cc/170 EGC)	ME-NBI EGD	90.91	91.18/90.64	–
Hirasawa T *et al.* [27]	2018	Detection of early and advanced gastric cancers	R	CNN system based on SSD	Cafe deep-learning framework	2,639 GC lesions	13,584 images	Non-ME, WLE/CE/NBI EGD	77 GG from 69 consecutive pts	2,296 images	WLE EGD		92.2; 98.6% detection rate—lesions larger than 6 mm	–
Horiuchi Y *et al.* [21]	2019	Diagnosis of EGC (differentiating GC vs gastritis)	R	22-layer CNN (GoogLeNet), trained using backpropagation	Cafe deep-learning framework		1,492 EGC/1,078 gastritis images	ME-NBI EGD		151 EGC and 107 gastritis	ME-NBI	85.3 EGD	95.4/71.0	0.852
Ishioka M *et al.* [120]	2019	Real-time detection of gastric cancer from video images	P, pilot	CNN system based on SSD	Cafe deep-learning framework	2,639 GC lesions [see 39]	13,584 still images [see 39]	Non-ME, WLE/CE/NBI EGD	68 ESD procedures for EGC in 62 pts	Video images	Video EGD		94.1	–
Kubota K *et al.* [121]	2012	Diagnosis of depth of invasion in gastric cancer	R	ANN trained using backpropagation	10-time CV	– 902 images					WLE EGD	77.2, 49.1, 51.0, and 55.3 for T1-4 staging		–
Zhu Y *et al.* [22]	2019	Diagnosis of depth of invasion in gastric cancer (mucosa/SM1/deeper than SM1)	R	Transfer learning methodology, based on a pretrained CNN architecture (ResNet50)	Adam optimizer		Training data set : 632 images, data augmentation → 5,056; validation data set: 158 images→1,264 images (augmentation)	WLE EGD		Test data set: 203 images	WLE EGD	89.16	76.47/95.56	0.94
Luo H *et al.* [125]	2019	AI for the diagnosis of upper gastrointestinal cancers	R/P	DL semantic segmentation model (encoder–decoder)	Internal, external, and prospective validation	For training and test GRAIDS: – 1,036,496 endoscopy images – 84,424 individuals					HD-WLE EGD	95.5 (internal validation); 92.7 (prospective set); 91.5–97.7 (5 external validation)	94.2/92.3 (prospective set)	0.966–0.990 (external validation data sets)

P, prospective study; R, retrospective study; EGD, esophagogastroduodenoscopy; DL, deep learning; CAD, computer-assisted diagnosis system; ANN, artificial neural network; CNN, convolutional neural network; IRL, iterative reinforced learning; CW, color wavelet; DSSD, deconvolutional SSD; mAP, mean average precision; RFSNN, Refined Feature Selection Using a Neural Network; WLE, white-light endoscopy; NBI, narrow-band imaging; HD, high definition; ME, magnifying endoscopy; IEE, image-enhanced endoscopy; BLI, blue-laser imaging; LCI, linked color imaging; G2LCM, Gabor: based gray-level co-occurrence matrix; CE, chromoendoscopy; GC, gastric cancer; EGC, early gastric cancer; AIM, acetic acid-indigo carmine chromoendoscopy; SIFT, scale-invariant feature transform; VS, classification system is based on microvascular (V) and microsurface (S) patterns; MSVE, multispectral video endoscopy; ESD, endoscopic submucosal dissection; SM, submucosa; GRAIDS, Gastrointestinal Artificial Intelligence Diagnostic System.

## Discussion and perspectives

To overcome the reduction in the quality of gastroscopy due to the high workload and different expertise levels of endoscopists, real-time computer-assisted quality-improvement systems in upper endoscopy based on the systematic screening protocol for the stomach have emerged that reduce the rate of blind spots during the procedure and improve diagnostic accuracy. The newly developed systems are able to increase the visibility of the mucosa, and might be used in endoscopic training programs and as method of assessing the skills of endoscopists. The only drawback of such an approach might be the increase in the procedure time.

A major application of computer-assisted systems is to help to recognize the anatomical location of the lesion, representing the first main step in supporting the diagnosis of endoscopic lesions. The available systems demonstrated good performance in the recognition of anatomical location so they may be implemented in clinical practice in the near future.

Due to the difficulty of endoscopic follow-up of premalignant gastric lesions combined with tattooing to mark the location of the lesions, the development of gastric non-invasive biopsy surveillance by means of computer simulation and optical instruments has emerged. This experimental computer navigation system proved to need a shorter time to guide retargeting of the pathologic area vs tattooing, but still requires accuracy optimization of the method for clinical application.

AI algorithms have been studied for the classification of gastric precancerous lesions, gastric-polyp detection, and measurement showing promising results. Moreover, the addition of CAD during upper endoscopy might offer quick risk stratification of patients for the development of gastric cancer according to *H. pylori* status. This denotes significant importance in countries with a high incidence of gastric cancer and with endoscopic mass screening for this neoplasm, which leads to an increased burden for endoscopists. Also, several recent studies were able to identify, although with less accuracy, the category of patients with eradicated *H. pylori* infection, demonstrating a moderate association of gastric-cancer development.

The AI systems proved also to be associated with adequate performance for identification of premalignant conditions associated with *H. pylori* infection, such as the presence and severity of gastric atrophy, presence of intestinal metaplasia, and severity of inflammation. These encouraging performances result by achieving a higher diagnostic accuracy vs endoscopists and reducing the endoscopists’ workload.

Numerous studies using AI detection and classification algorithms in conjunction with different advanced IEE techniques such as CE, FICE, ME-NBI, BLI, and HSI, most of them trained and validated on stored endoscopic images, demonstrated comparable or better diagnostic performance scores for EGC than those of the expert endoscopists and significantly higher than those of non-experts. There is still a high number of false-positive cases, most of them consisting of gastritis lesions associated with marked alteration in mucosal coloration or architecture, localized atrophy, or intestinal metaplasia that might mimic neoplastic changes—an aspect that should be improved in the future. Nevertheless, the promising results obtained demonstrate that the AI systems are capable of distinguishing between EGC and gastritis with a high sensitivity and negative predictive value, so their use could be extremely helpful in gastric-cancer screening. Moreover, CAD systems were able to distinguish with high accuracy the neoplastic-invasion depth, thus reducing the number of surgical resections, and improving the quality of life and prognosis for those patients.

To implement these computer-assisted algorithms in the real-time diagnosis of gastric cancer, pilot studies using video endoscopic images were developed. Although fast detection capacity and a high diagnostic accuracy for gastric cancer have been demonstrated, there still remain problems in differentiating between EGC and background gastritis.

We can state that, in current practice, AI detection is more important than the AI classification task, because tissue biopsy remains the gold standard whereas optical biopsy has still a limited accuracy. We should assume that, if a discrete neoplastic lesion had not been detected at all, then the optical biopsy would not have been done. Therefore, the most significant application of AI in the assessment of upper-GI lesions is the detection of early cancer and, to some extent, the depth prediction, although it is a challenging task.

There are several ongoing clinical trials assessing the value of AI methods in enhancing real-time upper-endoscopy quality control, EGC detection, and mucosal invasion, which will hopefully lead to a significant improvement in this emerging technology (Table 2). Moreover, AI software is currently incorporated in endoscopic devices that are routinely used in real-life settings to develop a CAD model for improving the diagnosis of upper-GI cancer.

**Table 2.** Clinical trials using AI for automatic quality control in upper endoscopy and diagnosis of gastric cancer

Status	Study title	Number ID/acronym	Study type	Conditions	Design/interventions	Outcomes	Target sample size (no. participants)	Region
Completed	A single-center, retrospective, open-label, randomized–controlled trial of AI vs expert endoscopists for diagnosis of gastric cancer	NCT04040374 [126]	Interventional	Gastric cancer	Diagnostic test: AI-based diagnosis Diagnostic test: the expert endoscopists-based diagnosis Trial included outpatients who underwent upper-GI endoscopy for GC screening and compared the diagnostic detection rate for GC of AI vs expert endoscopists	Primary: per-patient diagnosis of GC Secondary: number of images analysed for diagnosis of GC intersection over union (IOU) of gastric lesions diagnosis of AGC diagnosis of EGC agreement on image- and IOU-based diagnosis of GC between AI and expert endoscopists	500	Japan
Completed	Quality control of esophagogastroduodenoscopy using real-time EGD auxiliary system	NCT03883035 [127]	Interventional	Esophagogastroduodenoscopy	The development of an automatic quality-control system (EAS) and evaluation of its performance in: real-time measuring endoscopic inspection completeness, detecting GC, and evaluating gastric mucosal visibility	Primary: detection rate of GC Secondary: mean number of GC cases detected mean inspection completeness mean inspection time errors of EAS	1,060	China
Recruiting	Study on the effectiveness of gastroscope-operation quality control based on AI technology	NCT04384575 [128]	Observational	Gastric cancer	High workload, different skills of endoscopists → reduced quality of gastroscopy → missing lesions; trial assesses the value of AI methods for improving the quality of endoscopic diagnosis and treatment	Primary: accuracy, sensitivity, specificity	700	China
Recruiting	Development and validation of an AI-assisted system for predicting mucosal invasion of EGC	ChiCTR2000031111 [129]	Diagnostic test	Early gastric cancer	Patients with mucosal (T1a) EGC vs patients with non-mucosal (T1a) during observational interval The development of a DCNN system to detect mucosal EGC during upper endoscopy and improve its detection rate Endoscopic image collection of EGC and analysis of AI	Primary: detection rate of early mucosal (T1a) gastric cancer SEN, SPE, ACC, AUC of ROC	50 (EGC group) 50 (control group)	China
Recruiting	A prospective, multicenter research of deep-learning AI technology to assist gastroscopy in screening early gastric cancer	ChiCTR2000029001 [130]	Observational	Early gastric cancer	Group 1: AI-assisted gastroscopy Group 2: normal gastroscopy Assessing DL to: identify/record image in real time supervise guide endoscopists to: perform gastroscopy operations, photos, ↓missed inspection sites AI role in: improving EGC/precancerous-lesions detection rate improving the operation level of the endoscopists	Primary: EGC detection rate Secondary: operation time AI score the percent of patients being overlooked at each site the number of blind spots	6,000 (group 1) 6,000 (group 2)	China
Recruiting	A Deep Neural Network Improves the Quality of Endoscopic Examination	ChiCTR1800014809 [131]	Diagnostic test	Gastric cancer and colon cancer	Aim: to develop a DCNN system to detect EGC, colon cancer, and precancerous lesions without blind spots during endoscopy and improve the quality of endoscopy examination	Primary: blind-spot rate Secondary: the rate of blind spots in patients' photodocumentation completeness of photodocumentation generated by endoscopists/by AI in AI group/by AI and endoscopists in AI group Additional: time for gastroscopy	N/A	China
Recruiting	Comparison of painless, ultrafine and ordinary gastroscope endoangel-assisted blind spots: a prospective, single-blind, three-group, randomized, single-center trial	ChiCTR1900020920 [132]	Interventional	Rate of blind spots in gastroscopy	6 groups: EndoAngel Aim: to compare the tolerability of painless, ultrafine, and general gastroscopy	Primary: blind-spot rate Secondary: operation time comfort degree limb coordination vomiting reaction	437	China
Pending	Evaluating the effect of tandem EndoAngel on reducing the miss rate of gastric neoplasms: a prospective, single-center, randomized–controlled trial	ChiCTR2000034453 [133]	Observational	Gastric neoplasm	Group 1: AI first group Intervention: Patients first undertake AI-assisted upper endoscopy, then routine upper endoscopy Group 2: routine-first group Intervention: Patients first undertake routine upper endoscopy, then receive AI-assisted upper endoscopy Aim: to evaluate the effect of an AI system on reducing missed detection of GC	Primary: miss rate of GC Secondary: miss rate of high-risk gastric lesions miss rate of EGC detection rate of GC/high-risk gastric lesions the ratio of EGC to all GC the number of GC cases per procedure the number of high-risk gastric lesions per procedure observation time	870 (Group 1) 870 (Group 2)	China

GI, gastrointestinal; AI, artificial intelligence; DL, deep learning; DCNN, deep convolutional neural network; EGD, esophagogastroduodenoscopy; GC, gastric cancer; EGC, early gastric cancer; AGC, advanced gastric cancer; SEN, sensitivity; SPE, specificity; ACC, accuracy; AUC of ROC, area under the Receiver Operating Characteristics curve; EAS, automatic quality-control system; IOU, intersection over union.

## Conclusion

Although having a long and difficult way ahead and several limits to overcome, computer assistance for monitoring the quality of upper endoscopy and the detection of premalignant and malignant gastric lesions has shown promising results.

As an emerging field, refinement of AI algorithms and advancement in endoscopic devices will continue to increase the speed and accuracy of the detection of premalignant and malignant gastric lesions, leading to improvements in the quality of endoscopies whilst reducing the workload for physicians.

## Authors’ contributions

All authors contributed to the concept elaboration and literature-search strategy. D.C.L., M.F.A., and A.C.F. wrote the original draft of the article. Finally, all authors have provided critical review of the manuscript and approved the final version.
